# Supplementary material for: The Effects of Exogenous Salicylic Acid on Endogenous Phytohormone Status in Hordeum vulgare L. under Salt Stress
Source: Plants (Basel). 2022 Feb 24;11(5):618. doi: 10.3390/plants11050618 (PMC8912680; doi:10.3390/plants11050618)
Supplement: Supplementary file 1 [file plants-11-00618-s001.zip › plants-1591790-supplementary.pdf]

Table S1. Effects of salicylic acid (SA) pretreatment on endogenous cytokinin (CK) levels in the leaves and roots of *Hordeum vulgare* L. 'Ince-04' plants under control and saline conditions (150 and 300 mM). Values are mean ± SE (n = 6).

**Treatment**	**Leaf**						**Root**					
	**C**	**150**	**300**	**SA**	**SA150**	**SA300**	**C**	**150**	**300**	**SA**	**SA150**	**SA300**
tZ	2.20 ± 0.18	1.58 ± 0.04	0.96 ± 0.25	1.46 ± 0.06	0.68 ± 0.26	0.62 ± 0.05	0.62 ± 0.00	0.62 ± 0.00	0.48 ± 0.00	0.66 ± 0.01	0.37 ± 0.10	0.27 ± 0.00
tZR	0.55 ± 0.07	0.39 ± 0.03	0.27 ± 0.03	0.24 ± 0.00	0.40 ± 0.02	0.17 ± 0.01	0.74 ± 0.07	0.80 ± 0.08	0.48 ± 0.06	1.00 ± 0.08	0.20 ± 0.05	0.07 ± 0.00
tZ9G	8.49 ± 0.32	6.28 ± 0.06	5.62 ± 0.19	5.93 ± 0.14	6.47 ± 0.01	5.28 ± 0.00	10.78 ± 1.11	4.60 ± 1.55	6.47 ± 2.36	8.77 ± 0.48	15.41 ± 6.77	3.77 ± 0.63
tZOG	1.65 ± 0.20	1.62 ± 0.07	0.69 ± 0.07	1.02 ± 0.20	0.93 ± 0.19	0.64 ± 0.03	0.19 ± 0.01	*<LOD*	0.68 ± 0.06	*<LOD*	*<LOD*	*<LOD*
tZROG	1.67 ± 0.12	0.79 ± 0.12	0.50 ± 0.02	0.38 ± 0.09	1.31 ± 0.07	0.99 ± 0.03	0.70 ± 0.35	0.85 ± 0.00	0.54 ±0.03	1.31 ± 0.37	0.90 ± 0.07	0.65 ± 0.01
tZR'5MP	2.06 ± 0.07	0.67 ± 0.34	0.50 ± 0.26	0.57 ± 0.28	0.82 ± 0.11	0.43 ± 0.07	0.26 ± 0.00	0.43 ± 0.02	0.27 ± 0.05	0.58 ± 0.26	0.31 ± 0.07	0.25 ± 0.00
cZ	7.39 ± 1.38	9.18 ± 0.12	13.73 ± 1.03	8.11 ± 1.09	14.77 ± 0.02	1.79 ± 0.08	1.25 ± 0.02	1.88 ± 0.08	1.88 ± 0.23	1.65 ± 0.28	1.25 ± 0.03	0.35 ± 0.03
cZR	15.98 ± 3.11	19.94 ± 0.32	30.24 ± 0.64	16.19 ± 3.13	32.36 ± 0.19	4.00 ± 0.16	5.54 ± 1.84	7.50 ± 1.04	8.41 ± 0.89	7.62 ± 1.19	4.98 ± 0.05	0.42 ± 0.04
cZ9G	0.11 ± 0.00	0.05 ± 0.00	0.06 ± 0.00	0.05 ± 0.00	0.09 ± 0.02	0.07 ± 0.01	0.12 ± 0.05	0.09 ± 0.00	0.38 ± 0.09	0.10 ± 0.02	0.18 ± 0.06	0.09 ± 0.00
cZOG	75.35 ± 14.16	69.11 ± 1.37	84.91 ± 0.54	61.51 ± 3.44	62.35 ± 4.14	69.57 ± 2.50	41.98 ± 2.77	24.44 ± 1.66	29.46 ± 4.25	32.24 ± 1.13	42.73 ± 1.81	20.42 ± 0.33
cZROG	5.96 ± 1.41	5.29± 0.00	18.98 ± 1.12	4.34 ± 0.05	10.10 ± 0.27	17.16 ± 0.43	55.39 ± 2.37	69.60 ± 0.19	54.83 ± 0.39	57.63 ± 0.63	61.63 ± 0.46	28.86 ± 1.42
cZR'5MP	1.40 ± 0.20	1.47 ± 0.26	6.79 ± 0.30	1.44 ± 0.47	2.68 ± 0.12	4.66 ± 0.31	1.58 ± 0.67	8.02 ± 2.41	7.35 ± 0.74	4.75 ± 0.51	4.13 ± 0.57	0.99 ± 0.11
**iP**	0.98 ± 0.25	0.49 ± 0.03	0.64 ± 0.02	0.34 ± 0.00	0.84 ± 0.03	0.24 ± 0.00	0.15 ± 0.01	0.14 ±0.01	0.17 ± 0.00	0.29 ± 0.10	0.15 ± 0.02	0.13 ± 0.01
**iPR**	10.62 ± 2.20	13.13 ± 0.88	28.69 ± 0.83	12.21 ± 2.31	32.45 ± 1.09	2.70 ± 0.70	4.94 ± 0.88	7.75 ± 0.84	7.46 ± 0.06	11.51 ± 0.57	3.56 ± 0.19	0.82 ± 0.01
**iP9G**	0.41 ± 0.10	0.65 ± 0.06	0.47 ± 0.15	0.52 ± 0.09	0.45 ± 0.01	0.37 ± 0.03	2.78 ± 0.35	1.50 ± 0.11	1.43 ± 0.07	1.67 ± 0.08	2.35 ± 0.02	0.60 ± 0.00
**iPR'5MP**	0.83 ± 0.20	0.20 ± 0.03	0.80 ± 0.06	0.08 ± 0.01	0.47 ± 0.06	0.27 ± 0.05	0.45 ± 0.01	1.40 ± 0.14	0.74 ± 0.01	1.58 ± 0.35	0.64 ± 0.02	0.28 ± 0.05

C, control (0 mM NaCl and SA); SA, 0.5 mM salicylic acid; 150, 150 mM NaCl; 300, 300 mM NaCl; LOD, limit of detection; *t*Z, *trans*-Zeatin; *t*ZR, *trans*-Zeatin riboside; *t*Z9G, *trans*-Zeatin-9-glucoside; *t*ZOG, *trans*-Zeatin-O-glucoside; *t*ZROG, *trans*-zeatin-O-glucoside riboside; *t*ZR5′MP, *trans*-Zeatin-9-riboside-5′-monophosphate; *c*Z, *cis*-Zeatin; *c*ZR, *cis*-zeatin riboside; *c*Z9G, *cis*-Zeatin-9-glucoside; *c*ZOG, *cis*-Zeatin-O-glucoside; *c*ZROG, *cis*-Zeatin-O-glucoside riboside; *c*ZR5′MP, *cis*-Zeatin-9-riboside-5′-monophosphate; iP, N6-isopentenyladenine iPR, N6-isopentenyladenosine; iP9G, N6-isopentenyladenine-9-glucose; iPR5′MP, N6-isopentenyladenosine-5′-monophosphate
